# Follicular fluid and plasma lipidome profiling and associations towards embryonic development outcomes during ART treatment

**DOI:** 10.3389/fendo.2024.1464171

**Published:** 2024-12-26

**Authors:** Yingxin Celia Jiang, Qi Che, Xinmei Lu, Miao Liu, Yao Ye, Xiang Cao, Xushuo Li, Yanxia Zhan, Xi Dong, Yunfeng Cheng, Christopher O’Neill

**Affiliations:** ^1^ Charles Perkins Centre, The University of Sydney, Sydney, NSW, Australia; ^2^ Centenary Institute, The University of Sydney, Sydney, NSW, Australia; ^3^ Reproductive Medicine Centre, Zhongshan Hospital, Fudan University, Shanghai, China; ^4^ Center for Tumor Diagnosis & Therapy, Jinshan Hospital, Fudan University, Shanghai, China; ^5^ Department of Hematology, Zhongshan Hospital, Fudan University, Shanghai, China; ^6^ Institute of Clinical Science, Zhongshan Hospital, Fudan University, Shanghai, China; ^7^ Woolcock Institute for Medical Research, University of Technology, Sydney, NSW, Australia

**Keywords:** lipidomics, blastocyst formation, plasma, follicular fluid, LC-MS, oocyte developmental competence

## Abstract

**Introduction:**

It is well acknowledged that lipids assume a critical role in oocyte maturation and early embryonic metabolism, this study aimed to evaluate the relationship between the lipid composition of plasma and follicular fluid (FF), and the consequences of embryonic development. This study compared the lipidomic profiles of paired plasma and FF samples obtained from sixty-five Chinese women who underwent assisted reproductive technology (ART) treatments.

**Methods:**

Non-targeted lipidomics analysis.

**Result:**

Results not only indicated similarities in lipid composition between these biofluids, but also revealed a number of unique differences. The biomatrix distinction was found to be primarily driven by lipids belonging to the lysophosphatidylcholines (LPC), phosphatidylethanolamines (PE), ether PE, and triglyceride (TG) classes. In addition, specific species from these subclasses were discovered to be correlated with embryo development outcomes during ART. Notably, the composition of the fatty acyl chains appeared to play a crucial role in these associations. Furthermore, thirteen plasma lipid variables were identified, represented by Phosphatidylcholine 18:014:0 and PE P-18:020:1, which correlated with successful blastocyst formation (BF).

**Discussion:**

The present study demonstrated that FF has a distinctive lipid composition, setting it apart from plasma; and the association observed with embryonic development underscored an important role of lipid composition in the healthy development of oocytes.

## Introduction

Assisted reproductive technologies are employed as an effective treatment for certain types of infertility, while the rate of successful clinical pregnancies remains limited. This has encouraged the transfer of multiple embryos, resulting in a higher occurrence of multiple gestations and associated complications (1–3). To address this problem, single embryo transfer has been promoted as a superior alternative and is now a standard practice in ART treatment (4, 5). Embryos with high morphological scores are widely considered to correlate strongly with positive clinical outcomes and are therefore prioritised for transfer (6, 7). However, as not all retrieved oocytes yield such high-quality embryos (8), it is essential to evaluate embryos for strong developmental potential to optimise the treatment outcome.

Oocyte quality is a critical factor of component embryonic development (9). Lipids, along with glycogen, are crucial energy substrates for mammalian oocyte maturation and the early metabolic processes of early embryos (10). Beta-oxidation and related gene activation are observed during embryogenesis events, with notably high activity seen in the late morula and blastocyst stages; whereas, the inhibition of lipid metabolism significantly restricts embryonic growth (11). In metabolic syndromes represented by dyslipidaemia, both the quantity and quality of retrieved oocytes, as well as the pregnancy outcome after *in vitro* fertilisation and embryo transfer, were affected (12–14). In addition, aberrant in lipid profiles are observed in the circulation and other biological matrices of women with obesity or polycystic ovary syndrome (PCOS) (15, 16). While the importance of lipid metabolism in embryonic development is well-established, most research were conducted at the level of lipid classes. A comprehensive understanding of maternal lipid composition at the species level, and its relationship with assisted reproductive technology outcomes, such as blastocyst formation, remains elusive.

Lipidomics, a rapidly developing field in the omics discipline, focuses specifically on the analysis and measurement of lipid metabolism (17, 18). Lipids comprise a set of biomolecules, categorised based on their functional backbone and fatty acyl chain composition. Recent studies revealed that the structural differences of fatty acyl (FA) chains may contribute to the diversity of lipid physicochemical properties (19). The enhanced analytical power of lipidomics provides an opportunity for fingerprinting lipidomic profiles of different biomatrices and thus exploring the roles of individual lipid species in the content of health and disease.

This study sought to describe the compositional differences between plasma and follicular fluid lipidomes and to evaluate whether potential relationships between maternal lipids and embryonic development exist. Our findings indicated a clear segregation between plasma and FF lipid profiles, with the identification of a unique set of ART-related lipid species. Moreover, thirteen plasma lipids that, when considered collectively, predicted blastocyst formation rate. Altogether, these observations give a direction for future studies that explore the biological role of lipids in embryonic developmental competence.

## Method

### Study population

This study enrolled 82 Chinese women receiving conventional *in vitro* fertilisation (IVF) or intracytoplasmic sperm injection (ICSI) cycles between January 2022 and May 2022 at the reproductive centre of Zhongshan Hospital, Fudan University. The fertilisation method was selected based on semen analysis result obtained before the oocyte retrieval day. The study was approved by the Institutional Review Board of the hospital (#B2022-285), and written informed consent was obtained from all participants prior to their inclusion. Seventeen participants were excluded due to age criteria, incomplete demographic data, and/or missing either plasma or follicular fluid sample for lipidomic profiling, resulting in a final cohort number of sixty-five participants (**Supplementary Figure S1**). Baseline clinical characteristics of the excluded participants are presented in **Supplementary Table S1**.

A standard gonadotropin-releasing hormone agonist (buserelin) regimen was initiated on day 21 of a spontaneous menstrual cycle, or a standard antagonist regimen (ganirelix) was employed. Follicle-stimulating hormone (FSH) stimulation was initiated upon conformation of down-regulation by ultrasound and plasma oestradiol (E_2_) measurement through electrochemiluminescence immunoassay, or on day 2 or 3 of antagonist cycles. Patients received 150 to 225 IU of recombinant FSH, the dose determined by basal FSH level, BMI, and antral follicle count. One patient, for financial reasons, received human menopausal gonadotropin. Further stimulation doses were determined according to the standard criterion for follicular development, as determined by ultrasound and plasma E_2_ levels. When a minimum of three follicles attained a diameter of 18 mm, 6500 IU of Choriogonadotropin alfa was administered. Prior to general anaesthesia, a 2 ml of venous blood sample was collected utilising EDTA-treated anticoagulation tube. The sample was centrifuged at 1,500 g for 10 minutes, after which the supernatant was collected and preserved at -80 °C for later analysis.

Oocytes were retrieved by transvaginal ultrasound-guided needle aspiration of the follicles under deep conscious sedation. During oocyte retrieval, follicular fluid was aspirated from one to three follicles, ranging in size from 14 to 24 mm, for each participant. To ensure uncontaminated samples, only the midstream aspirate was collected. These samples were centrifuged at 10,000 g for 10 minutes. The supernatant was aliquoted and stored at -80 °C. Oocytes originating from the follicles of which fluid was collected were individually cultured to monitor their development. On day 1, the day following oocyte retrieval and insemination, each oocyte was evaluated for signs of fertilisation.

### Human embryo culture and scoring

Embryos were cultured under conditions of 37 °C and 6% CO_2_. Embryonic development and quality were evaluated by a team of embryologists according to standard morphological criteria. On day 3 post-fertilisation, cleavage-stage embryos were scored based on cell number, the degree of fragmentation, and symmetry. Fragmentation was scored from 0 to 4, corresponding to 0%, 1–9%, 10–20%, 21–50%, or >50% cytoplasmic fragmentation, respectively. Symmetry scores, ranging from 1 to 3, reflected the uniformity of cell shape, with a score of 1 indicating perfect symmetry (20). Embryos exhibiting more than 7 blastomeres with less than 20% fragmentation and the highest symmetry were classified as high-quality embryos and were preferentially selected for transfer. Blastocyst morphology was accessed utilising the Gardner blastocyst grading system (21), where a score was based on the degree of expansion and hatching status, as well as the characteristics of the inner cell mass and trophoectoderm.

### Chemicals and reagents

MS-grade methanol, MS-grade acetonitrile, and HPLC-grade 2-propanol were purchased from Thermo Fisher, CA, USA. HPLC-grade formic acid and HPLC-grade ammonium formate were purchased from Sigma-Aldrich (St. Louis, MO, United States).

### Sample preparation and lipid extraction

Samples, stored at the -80 °C, were thawed at 4 °C. For lipid extraction, a 20 μl aliquot of each subject’s follicular fluid and EDTA plasma samples was processed. 350 μl of precooled 2-propanol and an internal standard mixture were added to each sample and mixed for one minute. Quantification employed nine internal standards: cholesteryl ester (CE), ceramide (Cer), diglyceride (DG), LPC, lysophosphatidylethanolamine (LPE), phosphatidylcholines (PC), PE, sphingomyelin (SM), and TG, were used for quantification (Internal Standards Kit for Lipidyzer Platform 5040156; AB SCIEX, MA, USA). Following a 10-minutes incubation at room temperature, samples were kept overnight at −20°C to improve protein precipitation. The next day, samples were centrifuged at 13,800 g for 20 minutes utilising the Thermo Sorvall Legend Micro 21R Refrigerated Centrifuge (CA, USA). The supernatant of each sample was collected (200 μL) in a sample tube and stored at −80°C for additional MS analysis.

### LC-MS/MS method for lipid analysis

Follicular fluid and plasma lipidomes were analysed with an AB SCIEX QTRAP 5500 LC-MS/MS system. Extracted samples were introduced into a Waters Acquity UPLC BEH HILIC column (100 mm × 2.1 mm, 1.7 µm) equipped with a Waters Acquity UPLC BEH HILIC VanGuard Pre column (2.1 mm×5 mm, 1.7 µm). Mobile phase A consisted of 95% acetonitrile (acetonitrile: water, V: V, 95:5) with 10 mmol/L ammonium acetate, while mobile phase B consisted of 50% acetonitrile (acetonitrile: water, V: V, 50:50) with 10 mmol/L ammonium acetate. Ammonium hydroxide was titrated into both A and B phases until both reached pH of 8.2. A flow rate of 0.5 mL/min was employed. Gradient elution performed as follows: staring at 0.1% B phase, the proportion of B was raised to 20% over 10 minutes, then linearly increased to 98% from 10 minutes to 11 minutes. This 98% of B phase was held for 2 minutes before reverting to the initial condition of 0.1% B at 13.1 minutes. The 0.1% of B composition was held until 16 minutes. The positive electrospray ionisation mode injection volume was 2 μL, and the negative electrospray ionisation mode injection volume was 5 μL. N2 served as the nebulizing gas. The following parameters were applied: curtain gas: 35 psi, GS1: 50 psi, GS2: 60 psi, ion spray voltage: 5500V, declustering potential: 80V, entrance energy: 10V, collision energy: 50V.

### Lipid identification and abbreviation

Lipid identification and relative quantification were conducted utilising SCIEX OS software (AB SCIEX, MA, USA) and LipidSearch 4.0 (Thermo Fisher Scientific, CA, USA) (22). This software facilitates the direct analysis of raw data sourced from the UPLC-Orbitrap MS system (Thermo Fisher Scientific). Automated peak detection and annotation were carried out and aligned with the in-built database. After alignment, the retention time and molecular mass of each peak were manually checked to ensure accurate lipid identification. Measured peak intensities were exported and normalised to concentration according to the added amount of internal standard.

Lipid abbreviations adhere to the LIPID MAPS nomenclature (23). In general, annotation starts with a shorthand class nomenclature followed by the number of carbon atoms and double bonds present in the acyl chains. The separator (“_”) indicates the composition of two fatty acyl chains. In the case of phospholipids, the symbol “-O” denotes the presence of an alkyl ether bond, while the ‘-P’ signifies an O-alk-1-enyl-bond (also known as plasmalogen). Limited by MS resolution, triglycerides are presented as the observed acyl chain fragmentation following by the sum of the remaining two acyl chains, e.g., TG 44:1 as TG 16:1_28:0.

### Quality control and data processing

In conjunction with the sample sequence, a quality control (QC) sample was repeatedly measured to verify the accuracy and stability of the LC-MS performance. This QC sample was generated by combining equal volumes of randomly selected samples, thus representing all lipids present in the sample run. Following the search and alignment procedures, Pearson correlation analysis was applied across QC samples to confirm the repeatability of MS detection. Correlation coefficients over 0.9 denoted the benchmark for acceptable repeatability and stability. To address reproducibility, all peaks detected in the pooled QC samples were evaluated by calculating their relative standard deviation (RSD), with an RSD% exceeding 30% designated as the exclusion threshold (24). The outcomes of the data evaluation are shown in **Supplementary Figure S2**.

### Statistical analysis

Statistical analyses were conducted with either R (Version 4.3.2) or MetaboAnalyst (Version 6.0). Variable distributions were evaluated for normality employing the Shapiro-Wilk test. The *P*-values for normally distributed continuous data were determined with an unpaired t-test, while non-normally distributed continuous data were analysed with the non-parametric Mann-Whitney test. Categorical variables were analysed with Fisher’s exact test. All *P*-values are two-tailed, with *P*-value < 0.05 considered significant.

Lipid data was log10 transformed prior to differential abundance and correlation analyses. Comparison of lipid subclasses abundance was visualised with a hierarchical clustering heatmap. Differences in lipid species was depicted with a volcano plot, with a fold change greater than |2| and a false discovery rate (FDR) adjusted *P*-value less than 0.05 establishing the criteria for statistical significance.

To conduct multivariate statistical analysis, each log10-tansformed variable were standardized to a mean of zero and a standard deviation of one. Principal component analysis (PCA) was performed to access differences in lipidome abundance. Partial least squares discriminant analysis (PLS-DA) facilitated a comprehensive comparison between the two lipidomes, preserving the multi-dimensional nature of the lipidomics data. The validity of the PLS-DA model was confirmed through cross-validation and evaluated with the fraction of variable variation (R^2^Y) and the goodness of prediction (Q^2^Y). In general, a model is considered effective when both R^2^Y and Q^2^Y exceed 0.5. Premutation testing was conducted to prevent model overfitting. The contribution of each variable to differentiating between the plasma and FF lipidomes is measured by its variable importance in the projection (VIP) value. Lipids exhibiting a VIP value greater than1 and a *P*-value less than 0.05 were considered significant to the model and the group separation.

Spearman’s correlation analysis was conducted to evaluate the association between lipid profiles and four key ART outcomes. Plasma and FF lipidomes were analysed independently. In consideration of the large variable number, correlation heatmap include only pairs demonstrating at least a moderate correlation, specifically those with a correlation coefficient r greater than |0.35| and a *P*-value less than 0.05.

Predictive models were developed in R. Elastic-net regression was performed with the “glmnet” package (25). Untransformed lipidomic data was utilised for better interpretation. The elastic net, a combination of Ridge and Lasso regressions, is valuable for feature selection when the number of variables greatly exceeds the sample size. Lipidomics data were scaled and split into a “training set” (70%) and a “validation set” (the remaining 30%) with a random seed of 123. Models were trained on the “train data” across a spectrum of alpha parameters (α), with lambda parameter (λ) tuned through cross-validation. The alpha parameter, which balances the contributions of the two regressions, was selected based on the minimum root mean squared error (RMSE) computed against with the validation data; whereas lambda was determined by the smallest cross-validated mean squared error (MSE). After variable selection, multicollinearity was evaluated with variance inflation factors (VIF) and a Spearman correlation matrix. To minimise model redundancy, any variable with a VIF exceeding 5 was removed from the linear model.

## Results

### Demographics and clinical characteristics of the participants

EDTA plasma and follicular fluid samples were collected from 65 Chinese women of reproductive age during oocyte collection. Clinical characteristics and lipidomic measurements were compared across the treatment method groups, as fertilisation methods were not restricted during enrolment. Women receiving in IVF had a significantly higher baseline FSH level and fewer retrieved oocytes (**Table 1**). Despite these differences, the two groups shared certain commonalities. Moreover, unsupervised PCA indicated no significant difference in FF and plasma lipidome distribution between the two fertilisation methods (**Supplementary Figure S3**). Therefore, the participants and their data were merged for later analysis.

**Table 1:** Clinical characteristic comparison between subjects with different fertilisation methods (ICSI/IVF).

	ICSI (N=40)	IVF (N=25)	*P*-value
Female age (years)^#^	31.6 ± 3.71	31.3 ± 3.14	0.733^a^
Male age (years)^#^	33.7 ± 6.14	33.1 ± 4.60	0.908
Female BMI (kg/m^2^)^#^	22.1 ± 3.00	22.7 ± 3.64	0.495^a^
PCOS diagnosis (n)^##^	6 (15%)	1 (4%)	0.235^b^
Basal FSH (mIU/ml)	6.15 [5.40; 7.53]	7.20 [6.10; 8.80]	0.025^*^
Basal LH (mIU/ml)	5.40 [4.30; 6.55]	5.40 [4.00; 7.20]	0.696
Basal E_2_ (pmol/L)	116 [90.3; 162]	121 [95.9; 151]	0.751
Basal P_4_ (nmol/L)	0.60 [0.40; 0.83]	0.43 [0.20; 1.00]	0.452
No. of Oocyte retrieve	12.0 [8.00; 18.2]	10.0 [5.00; 12.0]	0.014^*^
No.of MI oocyte	0.00 [0.00; 1.00]	0.00 [0.00; 1.00]	0.935
No.of MII oocyte	8.00 [5.00; 12.0]	7.00 [4.00; 11.0]	0.457
2PN/MII rate	0.83 [0.71; 1.00]	0.88 [0.75; 1.00]	0.578
Normal fertilisation rate	0.59 [0.42; 0.72]	0.73 [0.55; 0.85]	0.065
Cleavage rate	1.00 [1.00; 1.00]	1.00 [1.00; 1.00]	0.977
High-quality embryo rate	0.38 [0.16; 0.66]	0.35 [0.07; 0.57]	0.669
Blastocyte formation rate	0.50 [0.08; 0.60]	0.28 [0.00; 0.63]	0.4

Nonparametric data are presented as median (interquartile range). (^#^) Normally distributed data as mean (SD). (^##^) categorical data as n (%). *P*-values: Mann-Whitney test as default. (^a^), unpaired t-test with two tails; (^b^), Fisher’s exact test. *is indicated *P*-value <0.05. BMI, body mass index; PCOS, polycystic ovary syndrome; FSH, follicle stimulating hormone; LH, luteinizing hormone; E2, oestradiol; P4, progesterone; MI, oocytes in metaphase I; MII, oocytes in metaphase II; 2PN, zygotes with two pronuclei.

### Distinct lipid profiles between human plasma and follicular fluid

**Figure 1:**
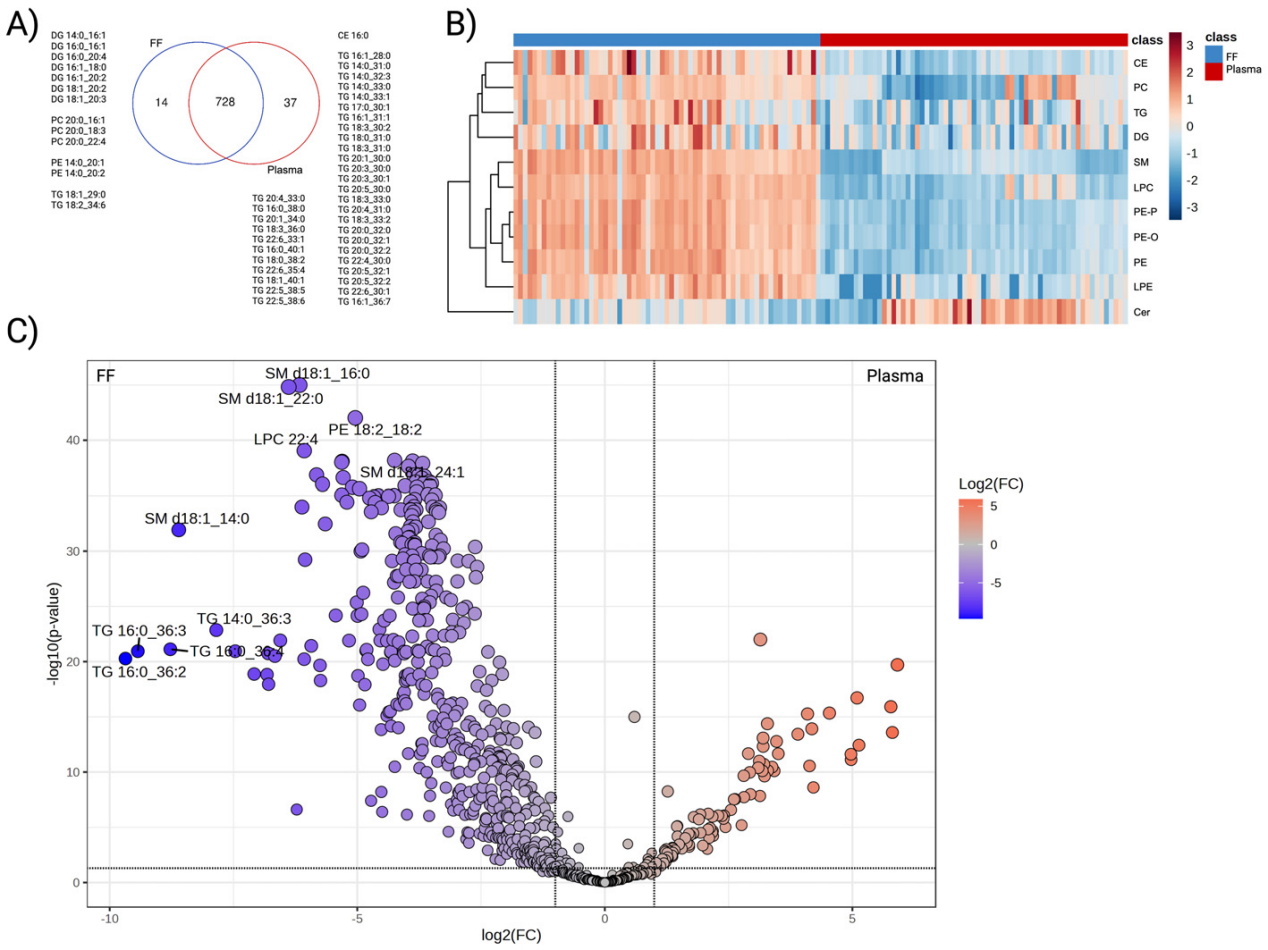
Differential abundance analysis of FF and plasma lipidome. **(A)** Venn diagram displaying the overlapped and unique species detected from the two biological matrices, **(B)** Hierarchical clustering heatmap representing the average abundance of the eleven detected subclasses. Paired comparisons were performed between FF (blue) and plasma (red). Lipid subclasses presented in rows with the log10-transformed abundance further standardised to Z-score. **(C)** Volcano plot that illustrated lipid species with differential expression patterns. Lipids that were enriched in FF were coloured in blue and plasma in red. Colour-filled dots indicated species with abundance difference > |2| folds and FDR adjusted *P*-value < 0.05.

A total of 742 FF lipids and 765 plasma lipids from eleven lipid classes, including CE, Cer, DG, LPC, LPE, PC, PE, PE-O, PE-P, SM, and TG were identified. Of these, 728 were common to both FF and plasma; however, a separate group of unique lipids was found in **Figure 1A**. FF-specific lipids comprised seven DGs, three PCs containing C20:0 fatty acid, two PEs containing C14:0, and two TGs. On the other hand, the lipid species identified in plasma were primarily TGs exhibiting a wide array of carbon chain lengths and degrees of unsaturation.

To compare plasma and FF lipidomes in the same individual, paired analyses were performed on the 728 shared species. Overall, a clear difference existed between the two biological matrices. As the heatmap illustrates, FF generally displayed greater abundance across all subclasses relative to plasma, with exception of Cer (**Figure 1B**). At the species level, 481 lipids demonstrated higher concentration in FF, whereas 102 lipids exhibited higher concentrations in plasma (FDR adjusted *P*-value <0.05) (**Figure 1C**). TG, PC, and PE were the most prevalent lipid classes enriched in FF, contributing 41.37%, 15.18%, and 13.10% of the differential, respectively. TG also constituted the largest subclass of plasma-enriched lipids (86.27%), followed by CE (4.90%), SM (3.92%), and DG (3.92%).

**Figure 2:**
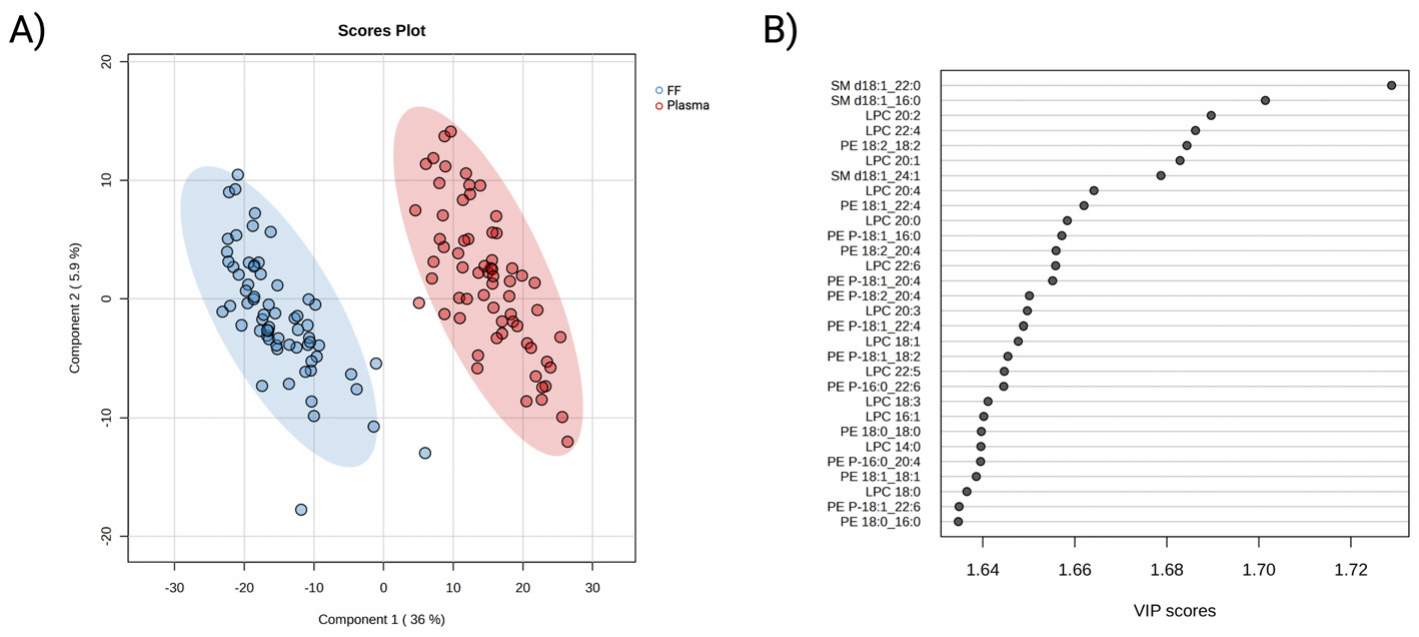
Multivariate analysis of FF and plasma lipidomic profiles. **(A)** PLS-DA 2D score plot of FF (blue) and plasma (red) lipidome composition from participants underwent ART treatment. **(B)** Identification of top 30 important variables that contributing to the distinguishment of the biofluid lipidomes. The contribution of individual lipids is measured by variable importance projection score (VIP), where a VIP >1 is considered as relevant to the model. A red-colour box on the right indicates a higher relative concentration, while a blue box indicates a lower concentration in the presented group.

Subsequently, partial least squares-discriminant analysis was employed to visually differentiate the two lipidomes. As presented in **Figure 2A**, the 2D score plot displayed clear segregation between the FF and plasma lipidome. Further cross-validation and permutation tests confirmed the classification result was neither overfitting nor random (**Supplementary Figure S4**). The VIP score measures a variable’s contribution to the model. A total of 313 lipids were determined to be involved in the differentiating between the two assigned biofluid groups (VIP >1 and *P*-value <0.05). Lipids belonging to the SM, LPC, PE, and PE-P subclasses were observed to be prevalent in the top 30 contributing variables (**Figure 2B**). Collectively, these results showed differences in plasma and FF lipid profiles, as well as the key role of maternal lipids on embryonic development in each biofluid separately.

### The correlation between lipid abundance and IVF outcomes

**Figure 3:**
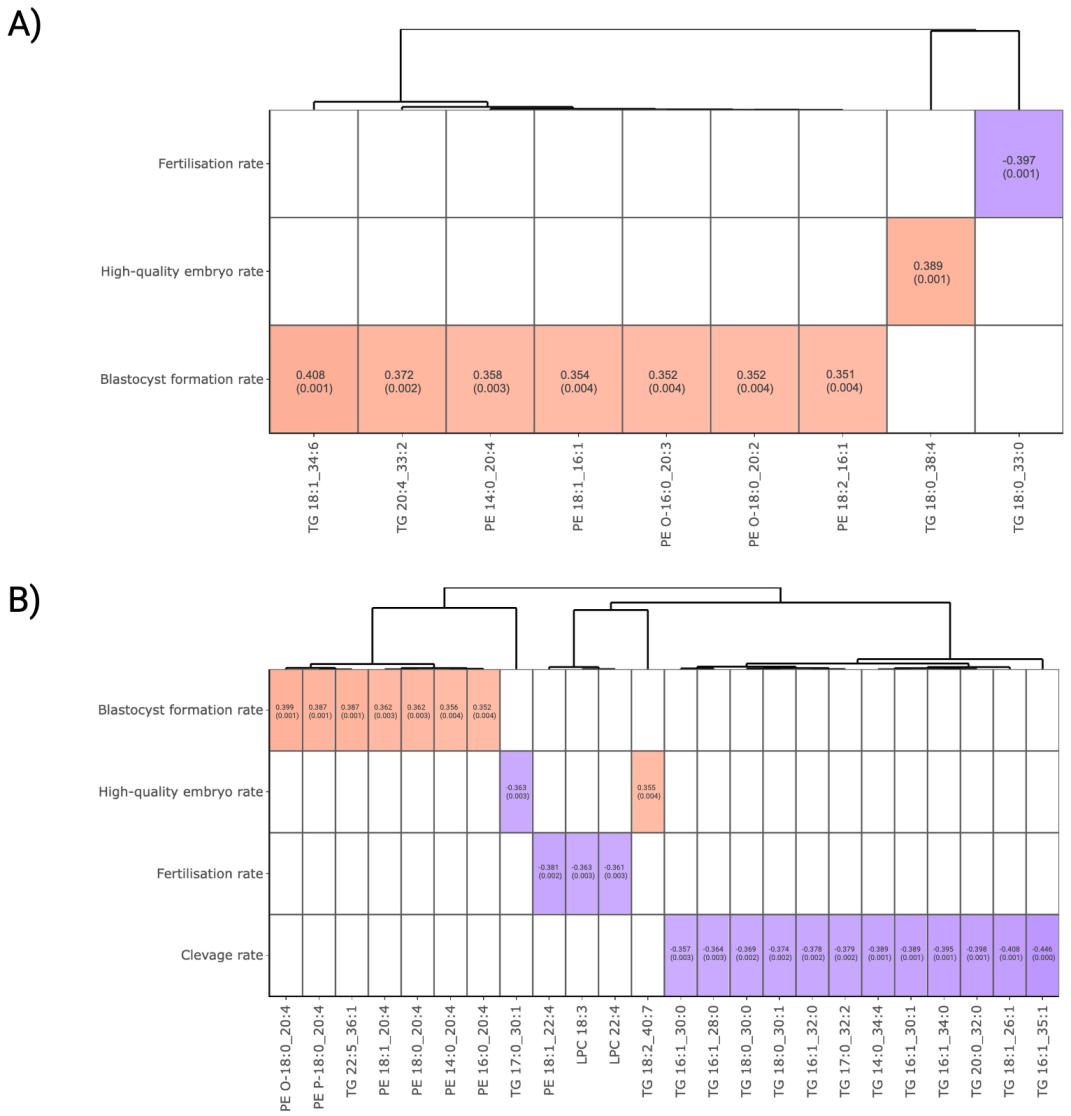
Correlation analysis between lipid abundance and ART outcomes. **(A)** FF and **(B)** plasma lipids that surpassed a threshold of Spearman’s correlation coefficient >|0.35| and *P*-value < 0.05 for visualisation. Lipids were presented in column and ordered according to the Ward clustering method. Red and blue boxes in heatmaps represent positive and negative correlation, respectively. The number in each cell indicates the correlation coefficient and *P*-value. The white colour indicates comparison pairs did not meet the displaying criteria.

To explore potential correlation between lipids and ART outcomes, Spearman correlations were performed utilising FF and plasma lipidomics data. **Figure 3A** presents the relationships between FF lipid abundance and embryonic development outcomes. By setting a threshold of a moderate correlation at correlation coefficient r >|0.35| and *P*-value <0.05, TG 18:1_34:6, TG 20:4_33:2, PE 14:0_20:4, PE 18:1_16:1, PE O-16:0_20:3, PE O-18:0_20:2 and PE 18:2_16:1 were discovered to be positively correlated with blastocyst formation. In addition, TG 18:0_38:4 was positively correlated with high-quality embryo formation, while a fully unsaturated TG 18:0_33:0 demonstrated a negative correlation with the fertilisation success rate.

In plasma, several C20:4 fatty acid-containing PE and ether-linked PE lipids, namely PE O-18:0_20:4, PE P-18:0_20:4, PE 18:1_20:4, PE 18:0_20:4, PE 14:0_20:4 and PE 16:0_20:4, demonstrated a positive correlation with BF rate (**Figure 3B**). While TG 22:5_36:1 and TG 18:2_40:7 exhibited a positive correlation with blastocyst and high-quality embryo formation, a panel of TGs containing 0 to 2 carbon double-bonds were found to be negatively associated with IVF outcomes. It was observed that the effect of TG on ART outcomes varies depending on their structural composition. In particular, polyunsaturated fatty acid (PUFA)-containing TGs tend to favour embryonic development more effectively than to those with a lower degree of unsaturation.

### Elastic net selecting lipids in predicting blastocyst formation outcomes

To identify lipids predictive of BF outcomes, we employed elastic-net regularisation regression to eliminate extraneous variables. The elastic net offers a compromise between the Lasso and Ridge regression, allowing variable selection while reduce multicollinearity and overfitting.

For the plasma model, an α of 0.3 produced in the lowest RMSE in the validation set when the optimal λ was its largest value (λ + 1 standard error). The subsequential process of turning the λ parameter was presented in **Supplementary Figure S5**. Among the 765 lipids, 14 were identified as effective estimators of BF. LPC 20:2 was removed to avoid multicollinearity of the model. The final elastic-net model composed 13 plasma lipids (**Supplementary Table S2**). Variables with a larger absolute coefficient value contributes more to the outcome variable. In this case, PE P-18:0_20:1 exhibited the strongest positive correlation with BF rate.; whereas PE O-16:0_20:3 and PC 18:0_14:0 were indicated to have the top ranking negative effect on blastocyst formation. After adjusting for other co-selected lipids and covariates, PC 18:0_14:0, PE P-18:0_20:1, and DG 18:2_20:5 were discovered to be independently correlated with blastocyst formation outcome (**Table 2**).

**Table 2:** Elastic-net regression model of plasma lipids after adjusted with covariates.

Variable	Coefficient	Std.error	t-value	*P*-value
(Intercept)	0.467	0.477	0.980	0.332
LPC 20:5	0.581	0.728	0.799	0.429
LPC 22:4	-0.070	0.391	-0.178	0.860
**PC 18:0_14:0**	-0.487	0.174	-2.790	0.008^*^
PE O-16:0_20:3	-1.054	0.877	-1.202	0.236
**PE P-18:0_20:1**	6.617	2.577	2.567	0.014^*^
PE P-18:0_20:4	0.008	0.010	0.840	0.405
**DG 18:2_20:5**	0.032	0.014	2.349	0.023^*^
TG 20:4_30:0	-0.057	0.309	-0.185	0.854
TG 20:3_32:1	0.047	0.148	0.320	0.751
TG 20:1_34:3	0.030	0.046	0.655	0.516
TG 18:1_37:4	0.021	0.309	0.069	0.945
TG 18:2_38:8	0.577	0.617	0.935	0.354
TG 20:4_38:5	0.240	0.130	1.845	0.071
Female age	-0.012	0.014	-0.863	0.393
Male age	0.001	0.009	0.161	0.873
Female BMI	-0.005	0.012	-0.405	0.687
Fertilization Method (ICSI=1)	0.145	0.078	1.853	0.070
PCOS history (Y=1)	-0.117	0.153	-0.763	0.449

Listed are the summary of elastic net regression model (α = 0.3, λ = lambda + 1SE) after adjusted with additional covariates. Male age, female age, female BMI, fertilization method and PCOS history were included as covariates. The regression model details are as following: multiple R-squared: 0.5861, adjusted R-squared: 0.4241, and *P*-value: 0.0002189. Lipid variables were scaled to mean of zero and standard deviation of one during model construction. Fertilization method was presented as IVF = 0/ICSI = 1, while PCOS history as No = 0/Yes = 1. Variables with a *P*-value <0.05 were ^*^ labelled and bolded for display.

Considering the lipids and their diverse biological functions, Spearman correlation was employed to illustrate their relationship. As depicted in **Supplementary Figure S5**, plasma lipids formed three groups. The levels of selected PC and LPC demonstrated strong correlations. Likewise, the three ether PE species exhibited a strong positive correlation. TGs, however, did not form a cohesive cluster, which indicates a potential difference in their synthesis activity. For the FF model, this approach did not yield any variables (Data not shown).

## Discussion

Assisted reproductive technology is now a widely recognised and effective treatment for infertility. Reaching the blastocyst stage is a critical step in pre-implantation embryonic development and a key observation point during *in vitro* embryo culture. To the best of our knowledge, this study is the first to explore the relationship between maternal biofluid lipid profiles and pre-implantation embryonic development. Lipidomic analyses of follicular fluid and plasma offer valuable information regarding individual’s metabolic states and can aid in identifying potential biomarkers.

It was known that the fundamental components of FF originate from plasma that traverses the blood-follicular barrier, as well as from cellular factors released by the granulosa and thecal cells (26). Our differential abundance analysis indicated a clear difference in the lipid composition of plasma and FF. LPC, PE, PE-P, and SM were identified as the most significant subclasses contributing to this difference. In addition, our findings indicates that FF is a “lipid-rich” biological matrix. Most measured lipid subclasses, except for Cer, were present at higher concentrations in FF compared to plasma. As a central molecule in the sphingolipid biosynthesis pathway, ceramide levels are tightly controlled under normal physiological conditions. PCOS and related metabolic conditions, such as obesity and diabetes, have been correlated with increased levels of circulating ceramide (27–30). Moreover, bovine studies have demonstrated that excess Cer hinders embryonic development at multiple pre-implantation stages (31, 32), potentially explaining the significantly lower ceramide levels observed in FF.

Next, we explore the relationship between lipid abundance and ART outcomes. In this study, we identified that plasma lysophosphatidylcholines, including LPC 18:3, LPC 22:4, and LPC 20:5, correlated negatively with fertilisation and blastocyst formation outcomes. Lysophosphatidylcholines are primarily generated from phosphatidylcholine (PC) through the action of Phospholipase A2, and thus their abundance is related (33). A growing body of research indicates that LPCs act as secondary messengers and play a role in the regulation of oxidative stress, inflammation, and apoptosis (33, 34). The pro-inflammatory effects of LPCs are well-reported in cardiovascular diseases. For instance, inhibiting NOX5 activity reduced LPC-induced oxidative stress in endothelial cells and monocyte adhesion, both of which are considered as early steps in the development of atherosclerotic lesions (35). Moreover, LPC has been shown to promote macrophage polarisation toward the pro-inflammatory M1 phenotype, contributing to vascular inflammation (36). While direct evidence is currently unavailable, a prior lipidomics study demonstrated that several LPC species, including LPC 18:3, were more abundant in women with PCOS (37). The observed inverse relationship between plasma LPCs suggests a systemic inflammatory condition that, accordingly, affects follicular development. However, in our data, FF did not display the expected negative effects of LPC. The reason for this discrepancy requires further explanations.

Our lipidomics analysis demonstrated that PE, PE-O, and PE-P promoted blastocyst development, with plasma PE P-18:0_20:1 exhibiting an independent positive correlation in our elastic-net model. PE is the second most prevalent phospholipid in mammalian cell membranes (38). In addition to its role as a structural component of the cell membrane, PE contributes to membrane fusion, mitochondrial homeostasis, and the biosynthesis of membrane proteins (39–42). Studies have confirmed that PE is essential for cytokinesis (43). Besides, knockout experiments demonstrated that disrupting PE synthesis pathways, through either PS decarboxylation or the CDP-ethanolamine pathway, resulted in a significant decrease in PE levels, abnormal mitochondrial function, and embryonic death in the affected mice (41, 44). Therefore, PE likely effects embryonic development through its involvement in cell membrane reconstruction and the modulation of mitochondrial function.

PE-O and PE-P (also known as PE plasmalogen) belong to a specialised class of phospholipids termed ether lipids (EL). While both processes an ethanolamine head group, ether PE biosynthesis utilises 1-*O*-alkyl glycerol-3-phosphate as a precursor and is reflected by the presence of an ether-linked fatty alcohol at the *sn*-1 position, rather than an ester-linked fatty acid (44). Due to this unique chemical structure, with its preserved carbon double bonds at *sn*-2 acyl chains, ELs are vulnerable to reactive oxygen species and function as antioxidants (45). Moreover, declined circulating plasmalogen levels has been correlated with both ageing and reduced fertility (46, 47), which is consistent with our modelling results. Collectively, the findings indicate that ELs may be involved in regulating vesicle-mediated processes, including fertilisation and embryo-maternal communication (48–50), while further analysis is necessary to confirm this hypothesis.

This study also indicated an inconsistent relationship between TGs and ART outcomes. Specifically, polyunsaturated fatty acid-containing TG species, including TG 18:1_34:6, TG 20:4_33:2, and TG 18:0_38:4 in follicular fluid (FF), as well as TG 22:5_36:1 in plasma, are correlated positively with the development of high-quality embryos and blastocysts. In contrast, less unsaturated plasma TGs demonstrated a negative correlation with embryo cleavage. Triglycerides represent the principle storage form of fatty acid, offering a crucial energy source for oocyte maturation and embryonic development as needed (51). Excessive circulating TG levels, however, has been correlated with increased oxidative stress (52), apoptosis (53), and abnormal oocyte development (54–56). It is important to note that the majority of previous research has concentrated on the TG synthesis pathway or utilised enzymatic assays to measure total TG levels.

Recent lipidomic analyses have demonstrated that the importance of chemical-physical composition, particularly fatty acid profiles, in dictating lipid biological function. Fatty acids, the fundamental components of lipids, can be classified as saturated or unsaturated based on the presence of double bonds. Numerous studies have reported the positive effects of long-chain polyunsaturated free fatty acids (FFA) on human health (57, 58). This is further corroborated by a longitudinal study demonstrating that circulating long-chain PUFA-containing TGs are correlated with a reduced risk of diabetes and related metabolic syndromes (59). In this study, we observed that PE and PE ether lipids positively correlating with blastocyst formation all contained a C20:4 fatty acyl chain. Arachidonic acid (AA, C20:4), an omega-6 polyunsaturated fatty acid derived from dietary source and abundant in the bloodstream, is rapidly cleared from circulation or incorporated into membrane phospholipid upon release (60). AA can also function as a precursor for inflammatory mediators, such as eicosanoids. High concentration of AA in FF has been demonstrated to affect follicle development and ovulation, possibly through AA-induced local inflammation (28, 61, 62). Therefore, a high concentration of AA-containing phospholipids may limit free AA accumulation, consequently impeding the creation of a pro-inflammatory environment during oocyte maturation.

Conversely, exposure to FFAs with low degrees of unsaturation is considered as toxic. High doses of saturated, monounsaturated, or diunsaturated FFAs have been demonstrated to cause lipotoxicity in human EndoC-βH1 beta-cells (63). Moreover, supplemental palmitic acid (C16:0) during *in vitro* maturation has been connected to mitochondrial dysfunction and disrupted human embryonic development (64, 65). Broadly, our findings support the concept that fatty acid composition is critical to lipid function. To the best of our knowledge, this study is among the few that have offered analysis of the relationship between human embryonic development and lipids at the species level.

Notwithstanding the contributions, several limitations should be acknowledged. First, the fertilisation method was not controlled in this study. While no apparent changes were observed in our study population, fertilisation methods are known to affect ART results, including the development of blastocysts. Speyer et al. demonstrated that IVF-derived embryos develop more rapidly without changes in blastocyst formation rate, compared to ICSI-derived embryos (66). Similar analyses had also been provided by several studies (67, 68). Another limitation of our study design is the changes in patients’ ovarian stimulation protocols. Certain studies indicate that superovulation drugs may affect the metabolite composition of follicular fluid (69). However, due to the small sample size, stratifying samples was not possible. Future studies with strict inclusion criteria and larger sample sizes are needed to confirm these results. Accordingly, changes in extraction solvents have been demonstrated to affect lipid yield, with the 2-propanol method producing lower sterol lipid concentrations compared to other solvent systems (70). While no changes were discovered in abundant phospholipids and sphingolipids, further research is necessary to verify these observations.

In conclusion, this study applied non-targeted lipidomic analysis to study the plasma and follicular fluid lipidomes and their relationship with pre-implantation embryo development. Our results indicated a clear differentiation between the lipidomic profiles of plasma and FF and identifying specific lipid sets correlated with ART results. Correlations with embryo development were noted in LPC, PC, PE, ether-PE and TG subclasses, with further associations based on individual lipid structures. Specifically, PUFA-containing TGs correlated positively with blastocyst development, whereas TGs with fewer double bonds demonstrated a negative effect on embryo cleavage and fertilisation. While the biosynthesis of individual lipid species remains poorly understood, our findings point to the significance of lipid metabolism in oocyte developmental potential and offered a direction for future studies.

## Data Availability

The raw data supporting the conclusions of this article will be made available by the authors, without undue reservation.
