# Supplementary material for: Follicular fluid and plasma lipidome profiling and associations towards embryonic development outcomes during ART treatment
Source: Front Endocrinol (Lausanne). 2024 Dec 26;15:1464171. doi: 10.3389/fendo.2024.1464171 (PMC11712041; doi:10.3389/fendo.2024.1464171)
Supplement: Supplementary file 3 [file DataSheet3.pdf]

Supplementary information

Follicular fluid and plasma lipidome profiling and associations towards embryonic development outcomes during ART treatment

Yingxin Celia Jiang, Qi Che, Xinmei Lu, Miao Liu, Ye Yao, Xiang Cao, Xushuo Li, Yanxia Zhan, Xi Dong*, Yunfeng Cheng*, Christopher O'Neill*

Supplementary Figure S1.

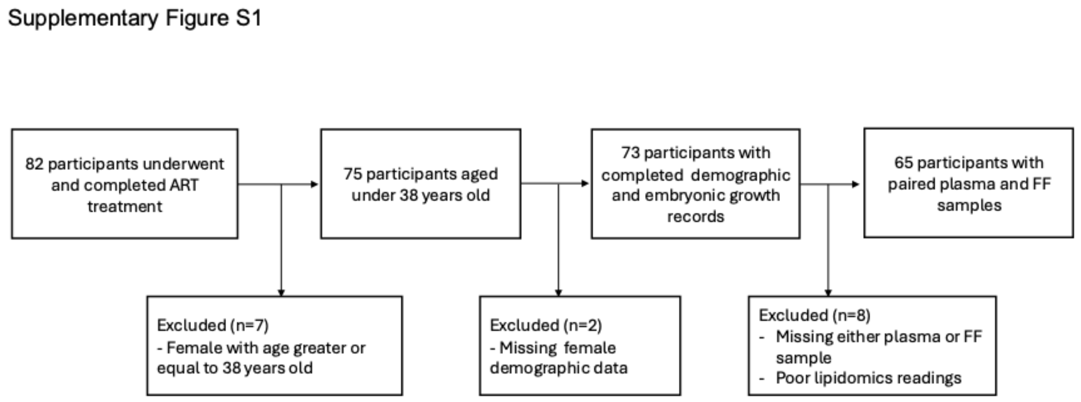

Schematic workflow inclusion and exclusion of the current study.

Supplementary Figure S2.

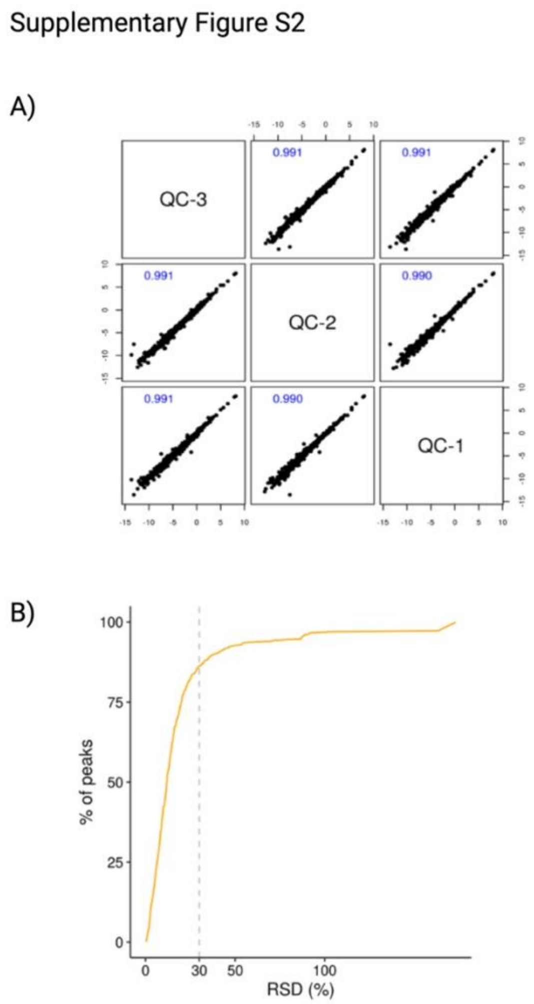

Evaluation of data repeatability and reproducibility. (A) Pearson correlation test based on the extracted peak intensity from the three repeated measurement of the same QC sample throughout the running sequence. (B) Distribution of the RSD% of lipid compounds peaks across QC measures. QC, quality control; RSD, relative standard deviation.

Supplementary Figure S3

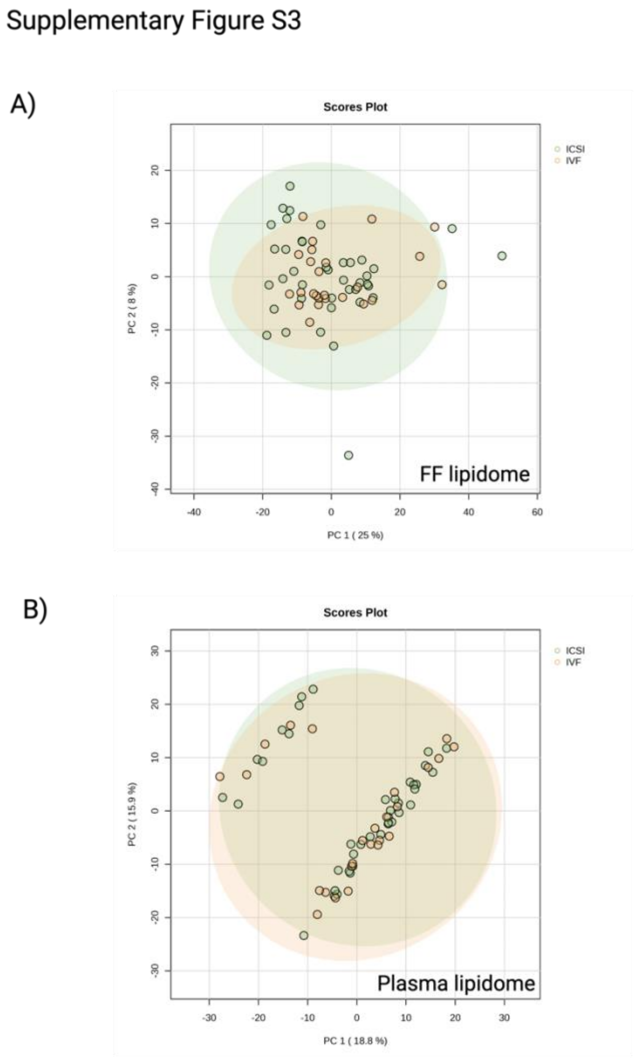

Unsupervised PCA comparison of (A) FF and (B) plasma lipidome between fertilization methods. No separation was observed between the two methods. Individual plots represent each participant, with IVF treatment colored in yellow and ICSI treatment in green. FF, follicular fluid, ICSI, Intracytoplasmic sperm injection; IVF, in vitro fertilization; PCA, Principal component analysis.

Supplementary Figure S4

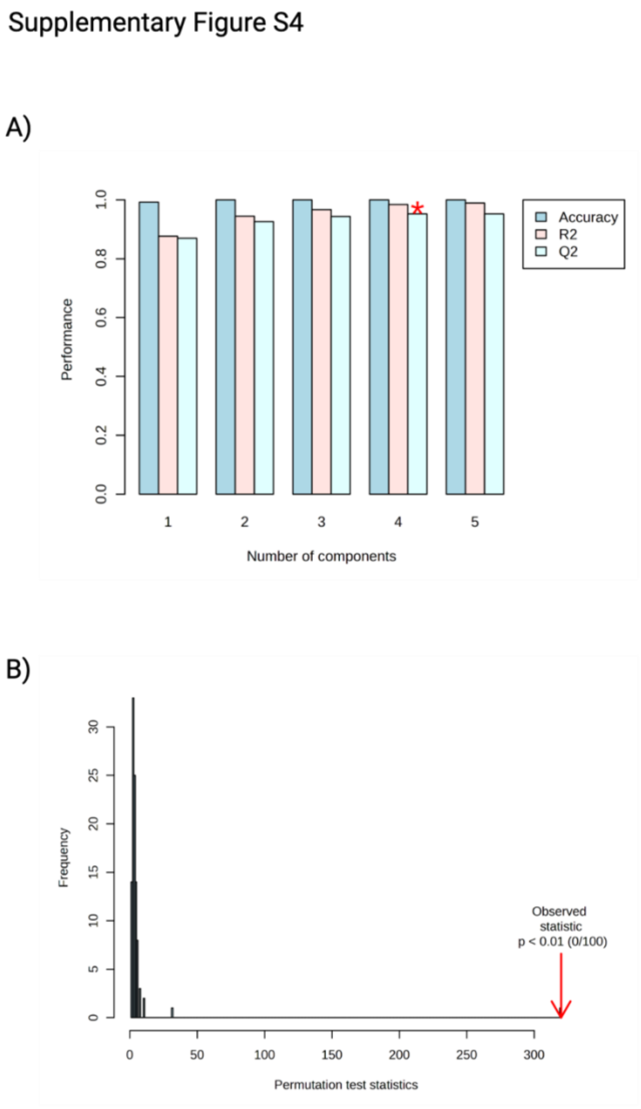

(A) 10-fold cross-validation and (B) 1000-times permutation test results of the PLS-DA model. The red asterisk represents the number of component where a highest Q^2Y is achieved. The highest PLS-DA R^2Y and Q^2Y of this model were 0.984 and 0.952 at the fourth component, respectively.

Supplementary Figure S5

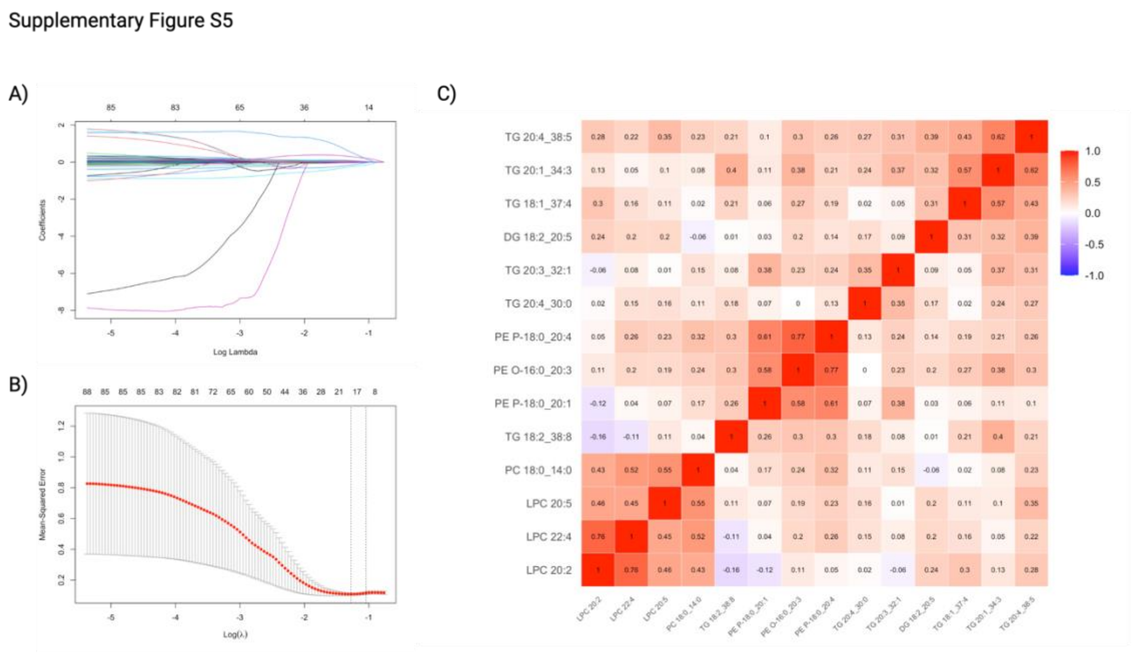

The outcome of elastic-net regression model when α = 0.3 and λ = lambda + 1SE. (A, B) Model construction and tunning of the penalty parameter lambda (λ). The λ threshold is determined based on individual variable coefficient and 10-fold cross-validation outcome. (C) Spearman correlation matrix of the 13 selected variables after the multicollinearity correction. Variable were clustered based on abundance similarity via the Ward.D method.

Supplementary Table S1. Baseline clinical characteristic comparison between the 65 enrolled subjects and the 17 excluded subjects.

	**Inclusion (N=65)**	**Exclusion (N=17)**	***P*-value**
**Female age (years)[#]**	31.5 ± 3.48	34.6 ± 5.71	**0.033*** [a]
**Male age (years)[#]**	33.5 ± 5.57	37.9 ± 8.20	**0.019***
Female BMI (kg/m^2)[#]	22.3 ± 3.25	23.2 ± 2.55	0.261 [a]
PCOS diagnosis (n)[##]	7 (10.8%)	1 (5.8%)	>0.999 [b]
Basal FSH (mIU/ml)	6.70 [5.45; 8.00]	6.90 [5.65; 9.95]	0.371
**Basal LH (mIU/ml)**	5.40 [4.00; 7.10]	4.10 [2.60; 5.45]	**0.023***
Basal E_2 (pmol/L)	120 [91.3; 162]	120 [81.9; 195]	0.881
Basal P_4 (nmol/L)	0.60 [0.30; 0.90]	0.50 [0.25; 0.80]	0.626
**No.of Oocyte retrieve**	11.0 [7.00; 17.0]	4.00 [2.00; 9.50]	**0.001***
**No.of MI oocyte**	0.00 [0.00; 1.00]	0.00 [0.00; 0.00]	**0.039***
**No.of MII oocyte**	8.00 [5.00; 12.0]	2.00 [1.50; 6.50]	**0.001***
2PN/MII rate	0.83 [0.70; 1.00]	0.91 [0.50; 1.00]	0.837
Normal fertilisation rate	0.61 [0.42; 0.77]	0.750 [0.415; 1.00]	0.248
Cleavage rate	1.00 [1.00; 1.00]	1.00 [1.00; 1.00]	0.626
High-quality embryo rate	0.37 [0.11; 0.65]	0.00 [0.00; 0.725]	0.341
**Blastocyte formation rate**	0.50 [0.00; 0.62]	0.00 [0.00; 0.185]	**0.004***

Note: Nonparametric data are presented as median (interquartile range). ([#]) Normally distributed female age, female BMI, as well as male age as mean (SD) for better comprehension. ([##]) categorical data as n (%). *P*-values: Mann-Whitney test as default. ([a]), unpaired t-test with two tails; ([b]), Fisher's exact test. *is indicated *P*-value <0.05. BMI, body mass index; PCOS, polycystic ovary syndrome; FSH, follicle stimulating hormone; LH, luteinizing hormone; E2, estradiol; P4, progesterone; MI, oocytes in metaphase I; MII, oocytes in metaphase II; 2PN, zygotes with two pronuclei.

Supplementary Table S2. Unadjusted elastic-net model of plasma lipids in predicting the outcome of blastocyst formation.

Variable	Coefficient	Std.error	t-value	*P*-value
(Intercept)	0.073	0.080	0.906	0.369
LPC 20:5	0.411	0.707	0.581	0.564
LPC 22:4	0.007	0.353	0.019	0.985
**PC 18:0_14:0**	-0.422	0.169	-2.498	**0.016***
PE O-16:0_20:3	-0.826	0.797	-1.036	0.305
**PE P-18:0_20:1**	5.726	2.244	2.552	**0.014***
PE P-18:0_20:4	0.009	0.009	0.928	0.358
DG 18:2_20:5	0.023	0.013	1.776	0.082
TG 20:4_30:0	0.090	0.293	0.307	0.760
TG 20:3_32:1	0.044	0.145	0.301	0.764
TG 20:1_34:3	0.032	0.040	0.791	0.433
TG 18:1_37:4	0.113	0.275	0.410	0.683
TG 18:2_38:8	0.576	0.608	0.948	0.348
TG 20:4_38:5	0.194	0.114	1.697	0.096

Note: Listed are the summary of elastic net regression model ($\alpha = 0.3$, λ = lambda + 1SE) using the continuous BF rate as outcome variable. The regression model details are as following: multiple R-squared: 0.543, adjusted R-squared: 0.4265, and *P*-value: 3.232e-05. Lipid variables were scaled to mean of zero and standard deviation of one during model construction. Variables with a *P*-value <0.05 were * labelled and bolded for display.
