# Supplementary figures and images for: Follicular fluid and plasma lipidome profiling and associations towards embryonic development outcomes during ART treatment
Source: Front Endocrinol (Lausanne). 2024 Dec 26;15:1464171. doi: 10.3389/fendo.2024.1464171 (PMC11712041; doi:10.3389/fendo.2024.1464171)

## Supplementary Figure S1

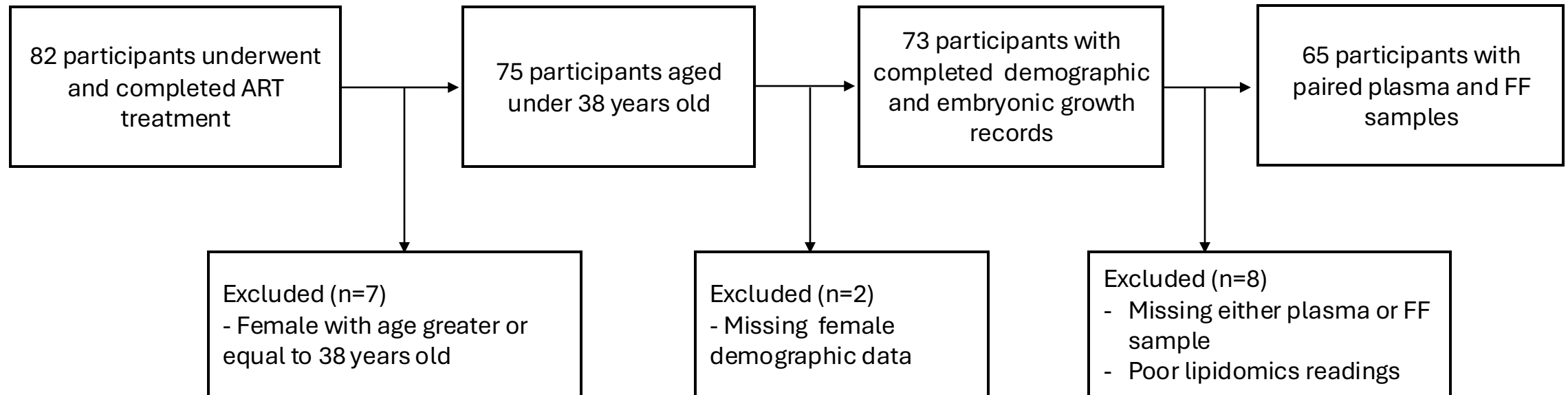

Supplement: Supplementary file 4 [file Image1.pdf]

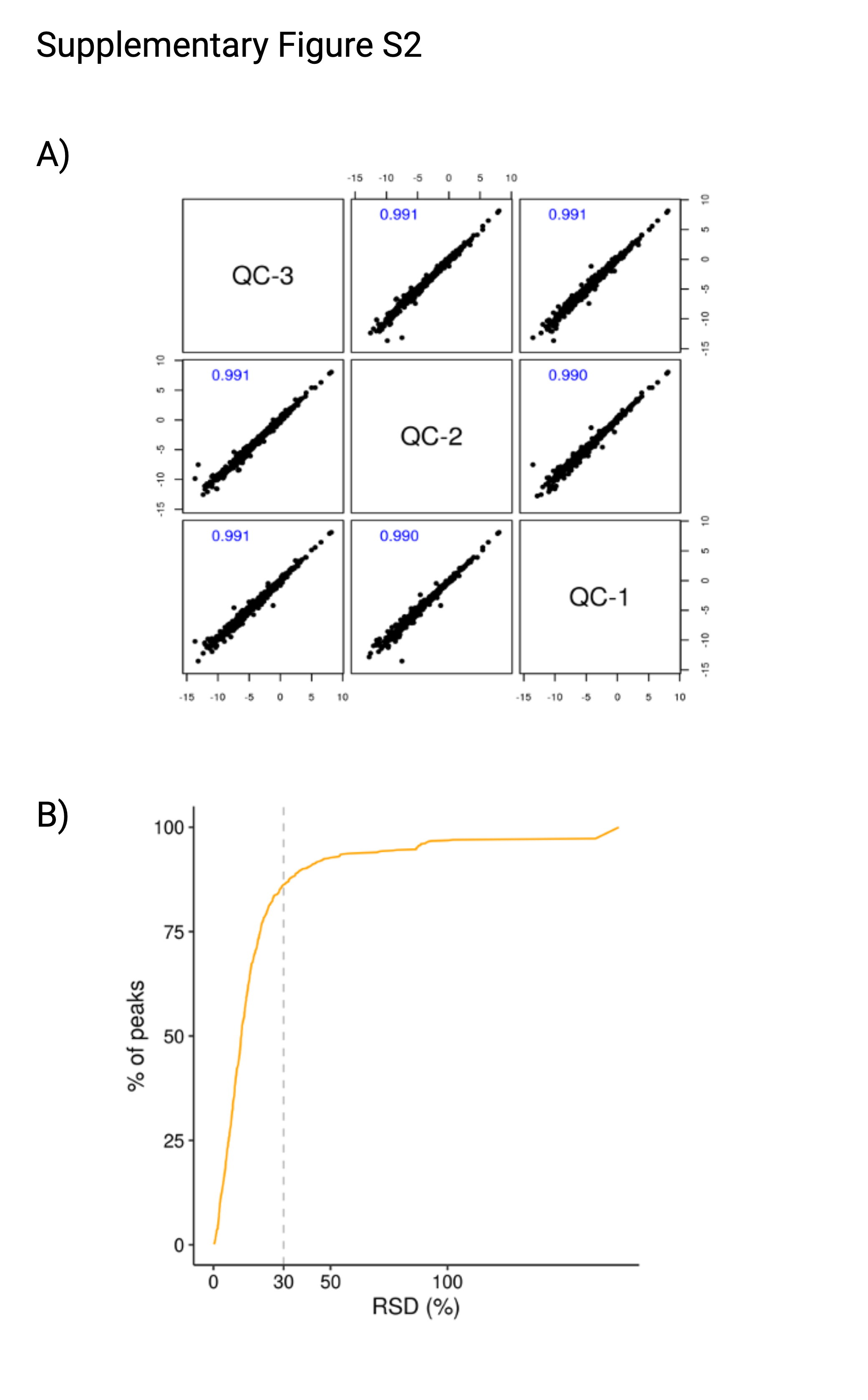

Supplement: Supplementary file 5 [file Image2.jpg]

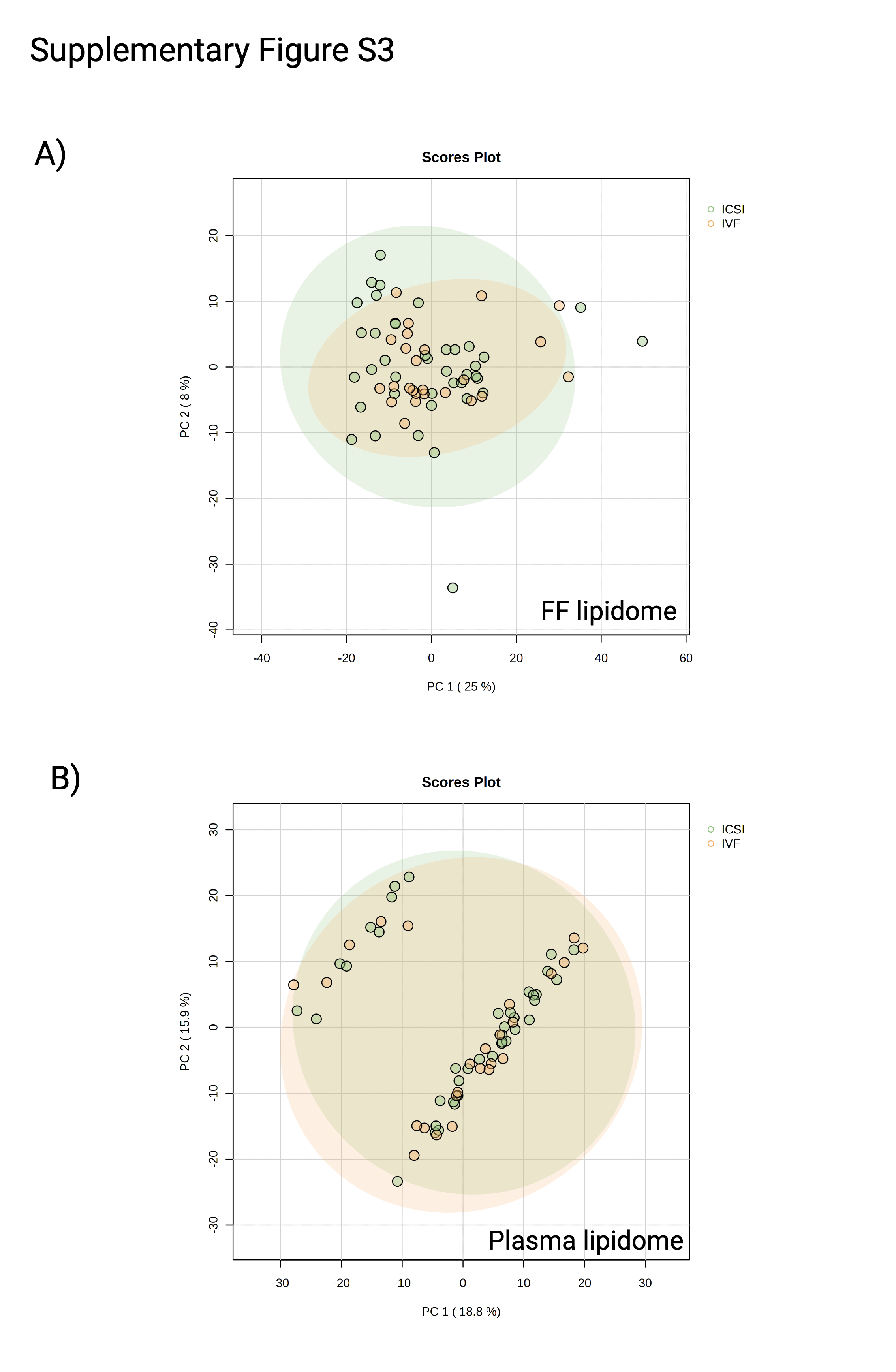

Supplement: Supplementary file 6 [file Image3.jpeg]

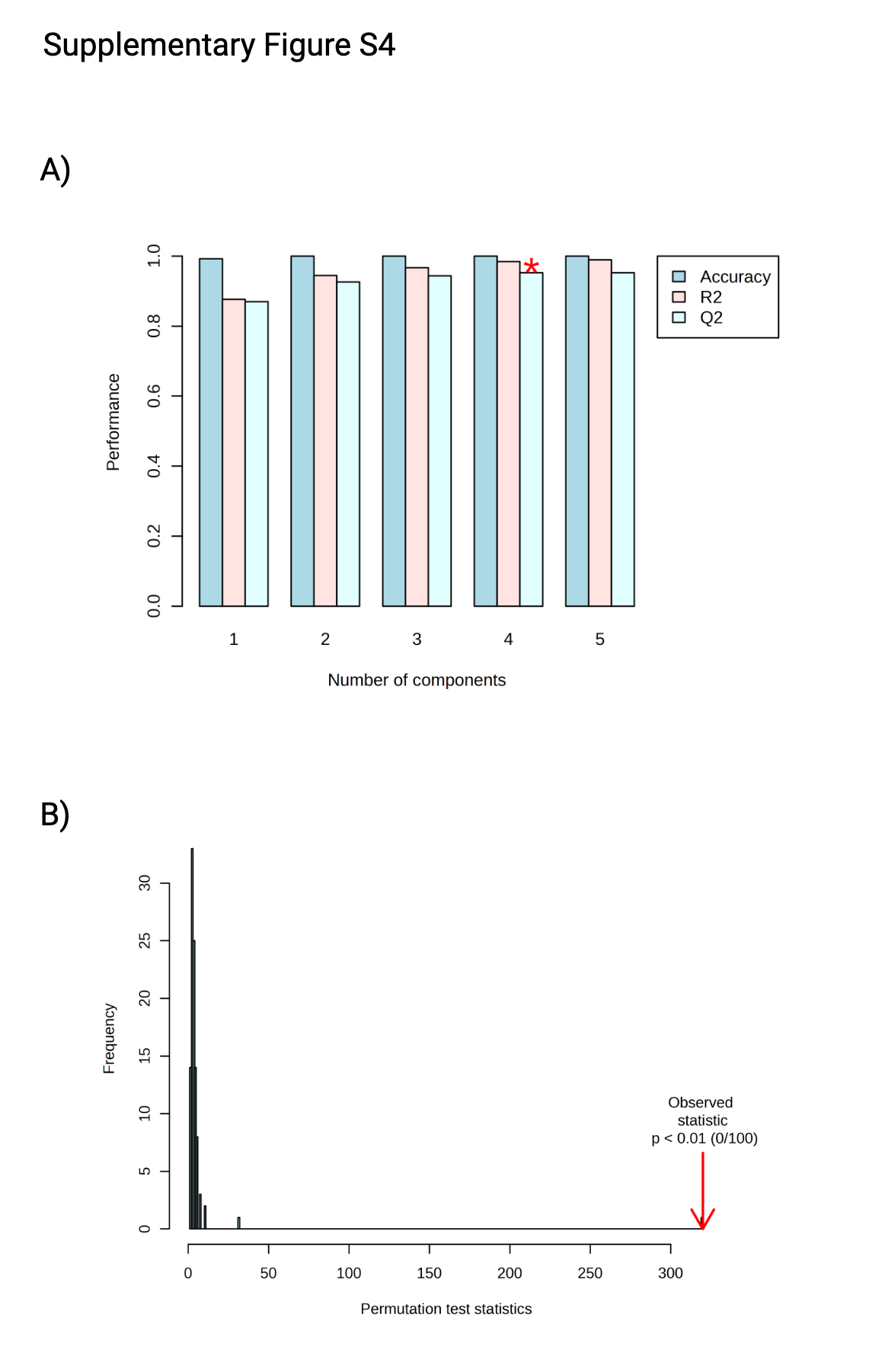

Supplement: Supplementary file 7 [file Image4.jpg]

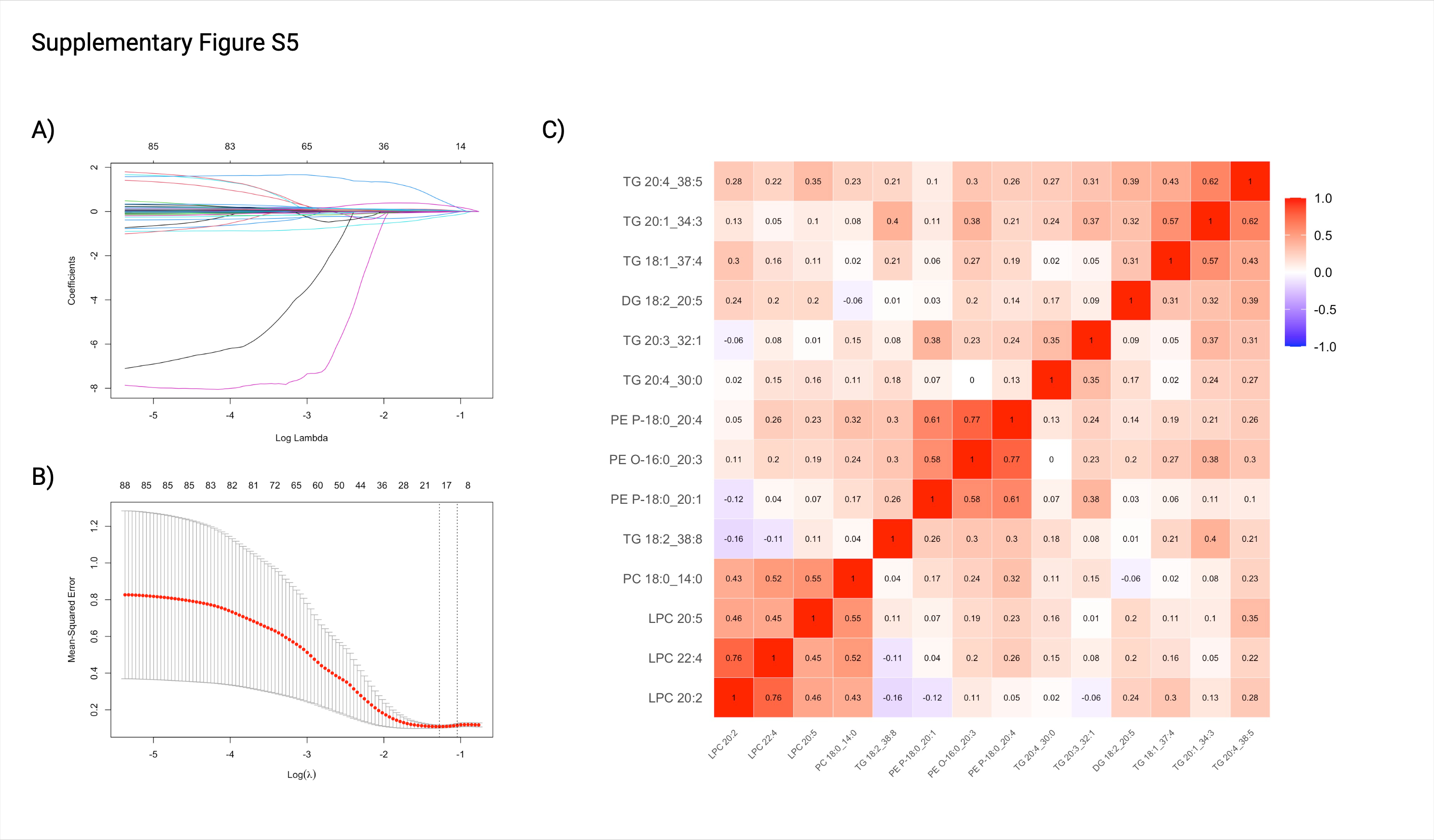

Supplement: Supplementary file 8 [file Image5.jpeg]
